# Development of a Method for Detecting *Alexandrium pacificum* Based on the Quantification of *sxtA4* by Chip-Based Digital PCR

**DOI:** 10.3390/toxins14020111

**Published:** 2022-02-02

**Authors:** Jun-Ho Hyung, Jinik Hwang, Seung-Joo Moon, Eun-Joo Kim, Dong-Wook Kim, Jaeyeon Park

**Affiliations:** 1 Environment and Resource Convergence Center, Advanced Institute of Convergence Technology, Suwon 16229, Korea; hjh1120@snu.ac.kr (J.-H.H.); sjmoon04@snu.ac.kr (S.-J.M.); kej1005@snu.ac.kr (E.-J.K.); 2 West Sea Fisheries Research Institute, National Institute of Fisheries Science, Incheon 22383, Korea; jinike12@korea.kr; 3 Food Safety and Processing Research Division, National Institute of Fisheries Science, Busan 46083, Korea; kimdw5791@korea.kr

**Keywords:** chip-based digital PCR, *Alexandrium pacificum*, paralytic shellfish toxin, saxitoxin biosynthesis gene, paralytic shellfish poisoning

## Abstract

*Alexandrium pacificum,* which produces the paralytic shellfish toxin (PST) saxitoxin (STX), is one of the causative species of paralytic shellfish poisoning outbreaks in coastal areas of Korea. In this study, we developed a chip-based digital PCR (dPCR) method for *A. pacificum* detection and tested it for monitoring in Jinhae-Masan Bay. Using the sequence of an *A. pacificum* strain isolated in 2017, species-specific primers targeting *sxtA4* (a STX biosynthesis-related gene) were designed and used in a dPCR, detecting 2.0 ± 0.24 gene copies per cell of *A. pacificum*. Cell abundance in field samples, estimated by a chip-based dPCR, was compared with the PST content, and measured using a mouse bioassay. A comparison with shellfish PST concentrations indicated that cell concentrations above 500 cells L^−1^, as measured using the dPCR assay, may cause shellfish PST concentrations to exceed the allowed limits for PSTs. Concordance rates between dPCR and PST results were 62.5% overall in 2018–2021, reaching a maximum of 91.7% in 2018–2019. The sensitivity of the dPCR assay was higher than that of microscopy and *sxtA4*-based qPCRs. Absolute quantification by chip-based dPCRs targeting *sxtA4* in *A*. *pacificum* exhibits potential as a complementary approach to mouse bioassay PST monitoring for the prevention of toxic blooms.

## 1. Introduction

Paralytic shellfish poisoning (PSP) outbreaks derived from harmful algal blooms result in human deaths and economic losses in the fisheries industry worldwide [1,2,3]. Blooms of *Alexandrium* species, *Gymnodinium catenatum*, and *Pyredinium bahamense,* which produce paralytic shellfish toxins (PSTs), caused these outbreaks [1,4,5,6]. In particular, *A*. *catenella*, *A*. *minutum*, and *A*. *pacificum*, which produce the PST saxitoxin (STX), and are distributed worldwide, are considered to be the primary causative species driving PSP outbreaks [1,7]. Toxic dinoflagellate blooms of *Alexandrium* species in Korea have occurred annually since the 1986 PSP outbreak in Busan, and the causative species in Jinhae-Masan Bay, where PST-producing blooms frequently occur, have been studied extensively [8,9,10,11]. Although *A*. *catenella* was thought to be responsible for the algal bloom events in Jinhae-Masan Bay over the past 30 years, Shin et al. [12] recently reviewed previous reports and reported that *A*. *pacificum* was one of the causative species of these events in the southern coastal waters of Korea, as well as *A*. *catenella,* based on resting cysts, the optimal temperature for growth, and phylogenetic analyses. However, since this re-evaluation, monitoring of the distribution of *A. pacificum* in Jinhae-Masan Bay has rarely been reported.

Currently, PST monitoring in the southern coastal waters of Korea is implemented by the Korean National Institute of Fisheries Science (KNIFS) based on mouse bioassays (MBA), a widely used method to measure PST concentrations [13,14]. However, this method is limited by potential ethical issues, high annual costs, low accuracy, and the time-consuming nature of the process; accordingly, attempts have been made to develop alternative methods [14,15,16,17,18,19]. In general, it is difficult to distinguish between *Alexandrium* species by light microscopy owing to the similarity in morphological features. Furthermore, rDNA-based qPCR assays tend to overestimate cell abundance in early and late bloom periods [19,20,21,22]. Recently, a quantitative PCR (qPCR) assay based on *sxtA,* which is a functional gene involved in PST biosynthesis, has been used for the detection of neurotoxin-producing dinoflagellates [19,23,24,25,26]. According to Murray et al. [19], assays targeting *sxtA* are generally more accurate than other established methods for quantifying and predicting toxic dinoflagellates.

The qPCR is widely used to estimate cell abundance and to monitor harmful algal species [27,28,29,30,31]. However, the results are affected by the standard curve and potential contamination (e.g., by salts, pigments, and other substances) [32,33,34]. Unlike the qPCR, a digital PCR (dPCR) is insensitive to potential PCR inhibitors and enables the absolute quantification of a target gene by counting the number of amplified DNA fragments in reaction samples using fluorescent signals; cell abundance in field samples can be estimated by chip-based dPCRs without standard curves as references [35,36]. Recently, Lee et al. [37] quantified the cells of *Margalefidinium* (*Cochlodinium*) *polykrikoides* in field samples by droplet dPCRs (ddPCRs) and measured the rDNA copy numbers per cell with high accuracy. In addition, species-specific gene copies targeting *A*. *affine*, *A*. *catenella*, and *A*. *pacificum* have been determined with high sensitivities by ddPCRs [14]. However, dPCR assays have not been applied to the monitoring of the causative species of PSP outbreaks.

In this study, we performed long-term monitoring (from 2018 to 2020) of the abundance of *A. pacificum* in samples from four locations in Jinhae-Masan Bay, Korea, by quantifying one of the genes, *sxtA4*, associated with STX biosynthesis, with a chip-based dPCR. Briefly, *A*. *pacificum* cells were isolated and identified by their morphological and molecular characteristics and the number of *sxtA4* gene copies per cell was quantified by a chip-based dPCR. Comparing the estimated cell count with the PST content at the same stations reported by the KNIFS, we obtained an optimal cell regulatory threshold for the dPCR assay to be used to determine if shellfish harvesting should be permitted. Hence, the dPCR assay is expected to complement traditional MBAs for detecting the early stages of blooms, and monitoring and preventing PSP outbreaks.

## 2. Results

### 2.1. Identification of the Masan Strain of Alexandrium pacificum

**Figure 1:**
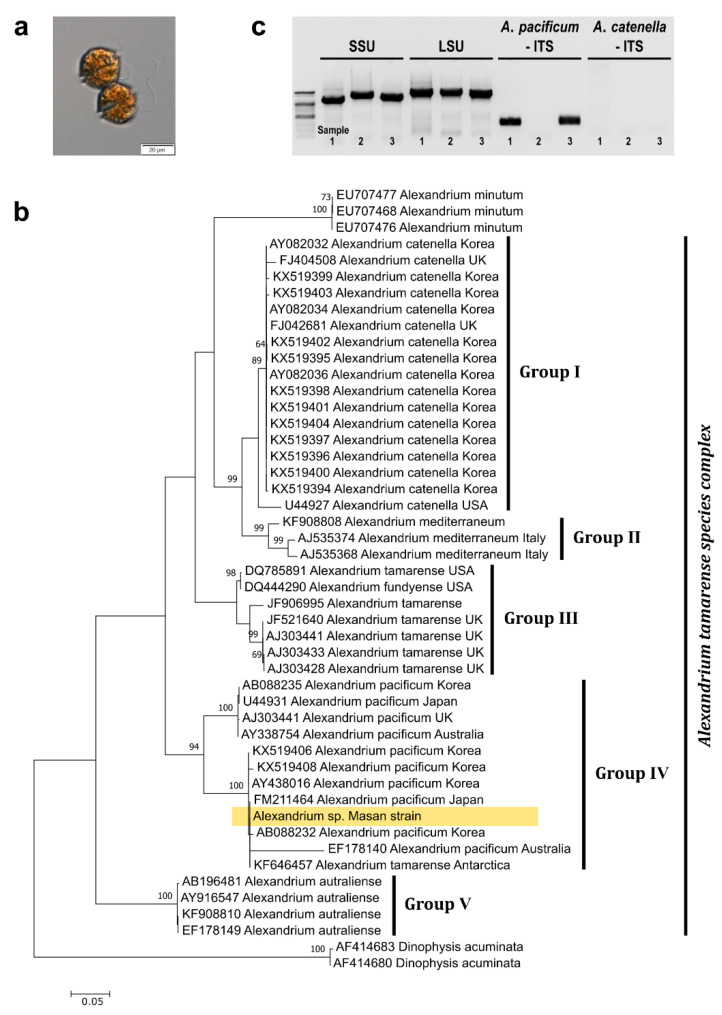
Taxonomic identification of the Masan strain of *A. pacificum*. (**a**) Light micrograph of cells; (**b**) phylogenetic analysis of *Alexandrium* strains using the maximum-likelihood (ML) method based on LSU rDNA sequences. ML bootstrap values (>60%) are indicated at the nodes and *Dinophysis* species were used as outgroups; (**c**) conventional PCR using ITS-specific primers for *A*. *pacificum* and *A*. *catenella*, as well as universal dinoflagellate primers for the SSU and LSU regions of rDNA, and three template samples: *A*. *pacificum* Masan strain from culture (lane 1) or GF/C glass microfiber filters (lane 3), and *A*. *minutum* as a negative control (lane 2). Scale bar = 20 µm (**a**) and the number of nucleotide substitutions per site (**b**).

Cells isolated from Jinhae-Masan Bay, in the southern coastal waters of Korea, were assigned to the genus *Alexandrium* based on light microscopy morphological analysis (Figure 1a). The Masan strain of *A. pacificum* was taxonomically identified based on a comparative sequence analysis of the large subunit (LSU) and internal transcribed spacer (ITS) regions of rDNA with that of other *Alexandrium* species deposited in GenBank. A maximum likelihood (ML) tree of LSU rDNA sequences containing 48 species revealed that the Masan strain clustered into Group IV, which includes *A*. *pacificum* in the *A. tamarense* species complex (Figure 1b). These findings were confirmed by a conventional PCR using species-specific ITS primers targeting *A*. *pacificum* and *A*. *catenella*, as well as universal dinoflagellate SSU and LSU rDNA primers as the positive control; *A*. *minutum* served as the negative template control. Evaluation of the amplified ITS products of the Masan strains of *A*. *pacificum* prepared from culture and from glass microfiber filtration indicated that they were both similar to that of *A*. *pacificum* (Figure 1c).

### 2.2. Quantifying Alexandrium pacificum Cells via Chip-Based dPCR Targeting sxtA4

**Figure 2:**
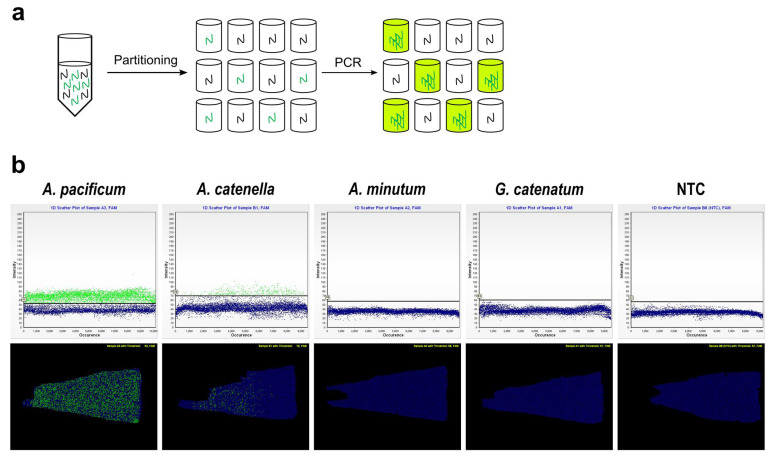
Chip-based digital PCR (dPCR) assay using a species-specific primer and probe set for *A*. *pacificum* targeting the *sxtA4* gene. (**a**) Schematic diagram of the experimental workflow for chip-based dPCR; (**b**) specificity test for the designed primer and probe set using the dPCR assay in the presence of *A*. *pacificum*, *A*. *catenella*, *A*. *minutum*, and *G*. *catenatum* with a known number of cells (i.e., 30,000 cells). Under optimized conditions with an annealing temperature of 62 °C, a 1D scatter plot (upper panel) of *A*. *pacificum* showed separation between *sxtA4*-positive (green) and *sxtA4*-negative (blue) signals, with a random distribution of cells on a position plot (lower panel). No template control (NTC) showed only blue background.

We implemented a chip-based dPCR in three steps: (1) partitioning DNA extracted from *A. pacificum* cells into multiple reaction wells; (2) PCR amplification using the *sxtA4*-specific probe and primer set designed in this study; and (3) measuring the proportions of positive and negative wells as detected by the fluorescent signal within a sample (Figure 2a).

To measure *sxtA4* gene copy numbers in the Masan strain of *A. pacificum*, partial *sxtA4* sequences (597 bp) in the cells were obtained by a conventional PCR; the analysis of the sequences showed a 100% identity with the *sxtA4* protein domain of *A*. *pacificum* (QRY28418), as determined using BLASTx. Based on the *sxtA4* fragments in the cells, we designed a *sxtA4*-specific primer and probe set, with an optimized annealing temperature of 62 °C, for *A*. *pacificum* to be used in a chip-based dPCR assay. The specificity of the primer and probe set was evaluated by applying the dPCR assay under optimized conditions to laboratory-cultured samples of *A*. *pacificum* Masan, *A*. *catenella*, *A*. *minutum*, and *G*. *catenatum*, setting the abundance of all measured species to 30,000 cells. For the dPCR assay, we obtained a sufficient amplification of *sxtA4* fragments in *A*. *pacificum,* as revealed by a 1D scatter plot (Figure 2b, upper panel), which showed a clear distinction between the positive and negative signals, and a position plot (Figure 2b, lower panel) displaying the distribution of amplified *sxtA4*. No amplified *sxtA4* products were detected for *A*. *minutum* or *G*. *catenatum*, whereas positive signals for some samples of *A*. *catenella* were observed. However, the distribution of positive targets on the position plots of *A*. *catenella* was biased toward the left, particularly when the annealing temperature for the dPCR assay was high, indicating that the primer and probe set showed poor performance for the amplification of *A*. *catenella* under the optimized conditions.

**Figure 3:**
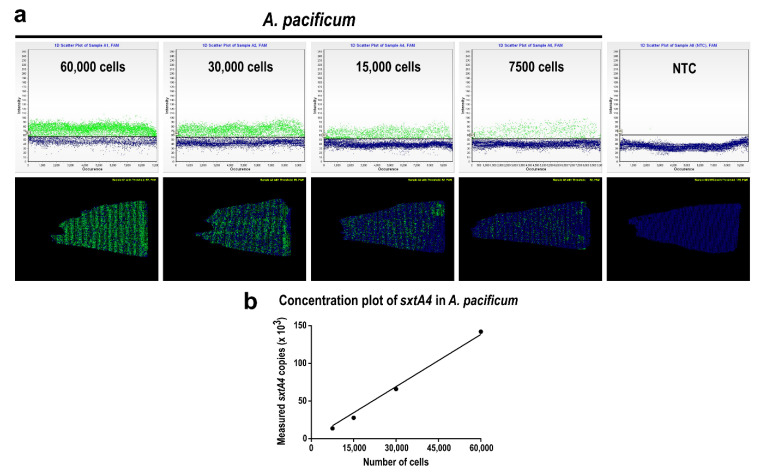
Quantification of *sxtA4* gene copy number in *A*. *pacificum* by chip-based digital PCR (dPCR). (**a**) 1D scatter plots and position plots for serially diluted samples of 60,000, 30,000, 15,000, and 7500 cells, and the no template control (NTC) for the dPCR assay; (**b**) copy number concentration plot generated based on the total number of *sxtA4* copies obtained for known *A*. *pacificum* cell numbers (*r*^2^ = 0.991 with *p* < 0.01). The plot was subsequently used to calculate the abundance of *A*. *pacificum* cells in field samples. The *sxtA4* gene copy number per cell of *A. pacificum* was 2.0 ± 0.24 (mean ± standard deviation, *n* = 20).

Using extracted gDNA containing known numbers of *A. pacificum* cells (i.e., 60,000, 30,000, 15,000, and 7500 cells), we measured the number of *sxtA4* gene copies in each sample by using the chip-based dPCR with the *sxtA4*-specific primer and probe set. As an end-point analysis, *sxtA4*-positive partitions showed green fluorescence, whereas *sxtA4*-negative partitions showed blue backgrounds (Figure 3). In the 1D scatter plots (upper panels), two distinct populations corresponding to positive and negative signals were clearly observed, and the number of positive partitions on the plots decreased in proportion to the reduction in known cell numbers of *A*. *pacificum* (Figure 3a). On position plots (lower panel in Figure 3), green-colored positive partitions were randomly positioned around the chip, and the number of signals decreased proportionally with the known cell numbers. Both the 1D scatter and position plot containing the no template control (NTC) showed only blue background signals. Analyses of the 1D scatter plots and position plots for the serially diluted samples confirmed that the absolute quantification of *sxtA4* gene copies in *A. pacificum* by chip-based dPCR was effective, and a threshold to determine the positive fluorescence in each partition was properly defined. We then quantified the number of *sxtA4* gene copies in 60,000, 30,000, 15,000, and 7500 cells, and plotted the total gene copies against the known cell counts. The total *sxtA4* copy number was significantly correlated with the number of cells (*r*^2^ = 0.991, *p* < 0.01), and the average *sxtA4* gene copy number per cell of *A. pacificum* was calculated to be 2.0 ± 0.24 (mean ± standard deviation, n = 20). This value was subsequently used to estimate the cell abundance of the toxic dinoflagellate in environmental samples (Figure 3b).

### 2.3. Comparison of Cell Abundance and PST Content in Field Samples

We collected 76 seawater samples from four locations: Jinhae (JH), Gusan (GS), Makpo (MP), and Neungpo (NP), in March and April from 2018 to 2021. Using these field samples, chip-based dPCR results and PST content, obtained from PSP monitoring reports by the KNIFS, were compared.

**Figure 4:**
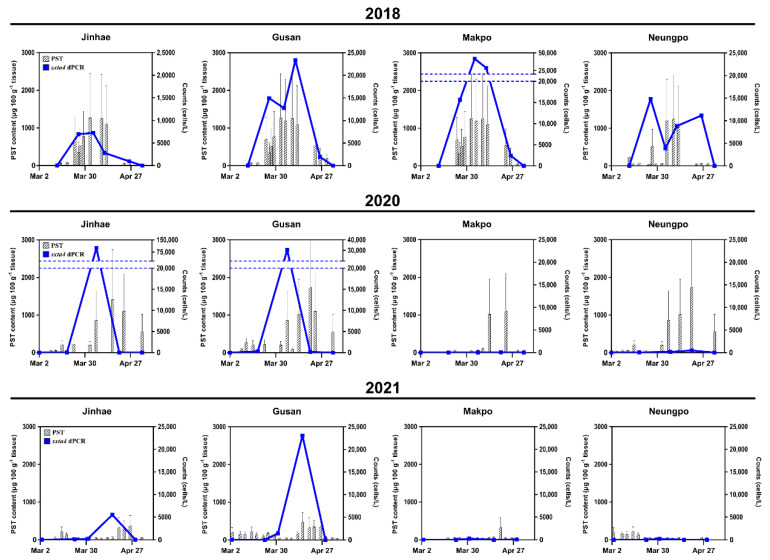
Comparison of cell abundance (cells L^−1^) obtained by the chip-based dPCR assay targeting *sxtA4* in *A*. *pacificum* cells and the PST content (μg 100 g^−1^ shellfish tissue) reported by the KNIFS at four sampling locations (Jinhae, Gusan, Makpo, and Neungpo) in March and April in 2018, 2020, and 2021. Blue line plots indicated cell concentrations of *A*. *pacificum* estimated by dPCR, and the PST content was presented in bar plots.

In March–April of 2018, *A*. *pacificum* cells were continuously detected at the four stations, and the peak abundance (cells L^−1^) for each location was 7250 (JH, 4 April), 23,342 (GS, 11 April), 41,826 (MP, 4 April), and 14,830 (NP, 26 March) (Figure 4, top row). The highest PST content (μg 100 g^−1^ shellfish tissue) at the same station, reported within 7 days after the date of the peak abundance, was 2424 (JH, 9 April), 2122 (GS, 12 April), 2424 (MP, 9 April), and 968 (NP, 27 March). The temporal patterns of *A*. *pacificum* occurrence detected by the dPCR assay during the period were highly similar to patterns of PST content at all four stations. In March–April of 2020, *A*. *pacificum* was detected at both JH and GS, but barely detected at MP and NP, except for the estimate of 468 cells L^−1^ at NP in April 2020 (Figure 4, middle row). At the JH and GS stations, peak abundances were 1.0 × 10^5^ (JH, 6 April) and 30,454 (GS, 6 April) cells L^−1^, and the highest PST contents were 2745 (JH, 16 April) and 1958 (GS, 13 April) μg 100 g^−1^ of shellfish tissue, as reported within 10 days after the date that the peak abundance was measured. The changes in cell abundance at JH and GS during this period were comparable to the reported changes in PST. 

The spatial distribution of *A*. *pacificum* at the four stations in March and April 2021 was similar to the distribution of cells in 2020. At JH and GS, the peak abundance of cells was 5488 (JH, 15 April) and 23,047 (GS, 15 April) cells L^−1^ (Figure 4). The highest PST content, reported within 7 days after the date that the peak abundance was detected, was 583 μg 100 g^−1^ of shellfish tissue at both JH and GS (19 April). PST content at MP, measured between March and April, was <80 μg 100 g^−1^ of shellfish tissue (except for 583 μg 100 g^−1^ of tissue on 19 April), similar to the occurrence of cell abundance at MP, which peaked at 255 cells L^−1^. In March 2021, PST measurements at the NP station ranged from 39 to 339 μg 100 g^−1^ of shellfish tissue, whereas *A*. *pacificum* cells were not detected, except for 224 cells L^−1^ on 31 March. In 2019, field sampling was performed only in March, and the dPCR assay at the four locations indicated a lack of *A*. *pacificum* (data not shown).

## 3. Discussion

The STX synthesis in *Alexandrium* species is initiated by the expression of the gene *sxtA* [38]. The s*xtA* mRNA transcripts in these species have both specific spliced leader sequences at the 5′ end and eukaryotic poly(A) tails at the 3′ end, indicating that the dinoflagellate transcripts are monocistronic, and *sxtA* is encoded in the nuclear genome of dinoflagellates [38]. The four catalytic domains of the *sxtA* gene form two isoforms (corresponding to the domains *sxtA1*–*sxtA4* and *sxtA1*–*sxtA3*) in *A*. *fundyense* [38]. The presence of the *sxtA4* gene in PST-producing species [38,39] provides a molecular basis for determining the abundance of toxic *Alexandrium* species.

We detected 2.0 ± 0.24 (n = 20) *sxtA4* copies per *A*. *pacificum* isolated from Masan in May 2017 by a chip-based dPCR using newly designed primers. Recently, Lee et al. [14] reported that the number of *sxtA4* copies per cell of *A. pacificum* isolated from Jangmok in February 2010, as determined by ddPCR, was 2.4 (n = 5), similar to the results of our study. Both of the strains were obtained from Jinhae-Masan Bay, where PST-producing blooms frequently occur due to *A. pacificum* [40]. A comparison of the spatial distribution estimated by quantifying *sxtA4* in each strain of *A. pacificum* is limited by the absence of monitoring results in the previous study.

**Figure 5:**
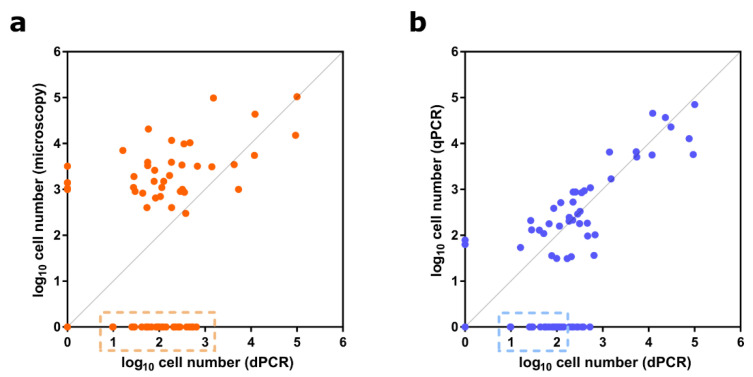
Cell number sensitivity of chip-based digital PCR (dPCR) targeting the *sxtA4* gene in *A*. *pacificum* compared to microscope cell counting and *sxtA4*-based quantitative PCR (qPCR). (**a**) Comparison between cell counts obtained by microscopy and cell estimates obtained by the dPCR assay. Microscope counting included all species in the genus *Alexandrium*; (**b**) comparison of chip-based dPCR and qPCR results for *A*. *pacificum* cell abundances based on the *sxtA4* gene. The boxed areas enclosed by dashed lines along the x-axis in (**a**) and (**b**) indicate the cell number detection limits for microscope counting and *sxtA4*-based qPCR, respectively. The measured cell numbers for each method were transformed to the log_10_ scale.

To verify the high sensitivity of a chip-based dPCR for detecting *A*. *pacificum*, we analyzed relationships between chip-based dPCRs and other detection methods, including qPCRs and microscopy, based on long-term monitoring results. The direct counting of cells by microscopy yielded overestimates of cell number compared to the abundance values obtained by chip-based dPCRs because microscopic identification of *Alexandrium* species was restricted to the genus level (Figure 5a). Using microscopy, the detection limit was less than ~1000 cells L^−1^ in field samples, whereas the chip-based dPCR assay enabled the detection of 10–1000 cells L^−1^ in environmental samples, as indicated in the boxed area in Figure 5a. Consistent with the above analysis, the detection limit for the *sxtA4*-based qPCR assay was 1000 cells L^−1^, but it was difficult to estimate the abundance in the range of 10–100 cells L^−1^ (Figure 5b). Considering that a monitoring system for PST-producing blooms is required for early detection and control, a chip-based dPCR targeting *sxtA4* with a high sensitivity for concentrations of < 1000 cells L^−1^ is expected to be effective in predicting the species distribution and preventing the downstream effects of blooms. 

**Figure 6:**
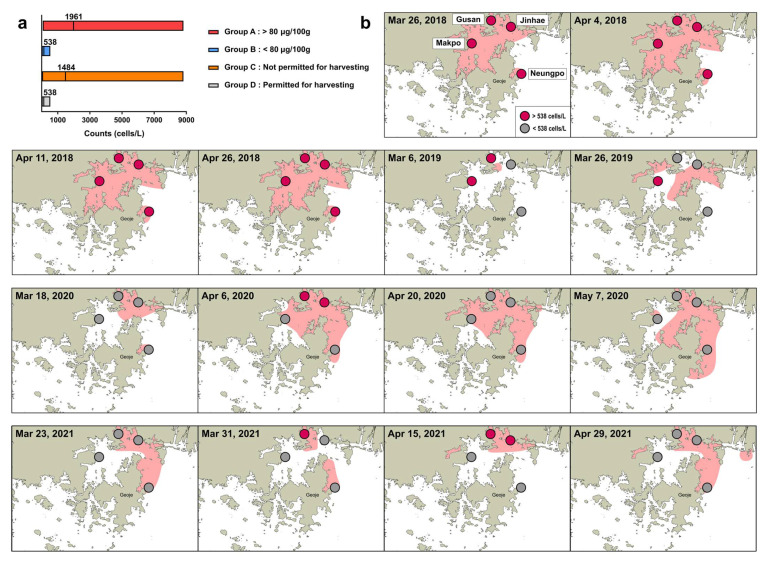
Optimization of the regulatory threshold for *A. pacificum* to determine whether shellfish harvesting should be permitted based on chip-based digital PCR (dPCR) targeting *sxtA4*. (**a**) Cell abundance estimated by the dPCR assay was evaluated in field samples collected in March and April from 2018 to 2021, and divided into four groups based on PST monitoring reports: Group A (n = 24), PST content at the station was higher than the regulatory sanitary threshold for PSTs (80 μg 100 g^−1^ shellfish tissue); Group B (n = 58), PST content at the station was less than the PST threshold; Group C (n = 35), shellfish harvesting in the area was regulated despite no detectable PST on the particular date; Group D (n = 48), shellfish harvesting in the area was permitted. (**b**) Comparison between *A*. *pacificum* monitoring using the regulatory threshold (538 cells L^−1^) based on the dPCR assay and PST monitoring based on the PST threshold at four localities (Jinhae, Gusan, Makpo, and Neungpo) in March and April from 2018 to 2021. Regulated areas for PSTs are displayed in red.

When the total PST content of shellfish tissue is higher than the regulatory threshold (80 μg 100 g^−1^ shellfish tissue), shellfish farms are not permitted to harvest any shellfish [13]. In this study, we optimized the cell regulatory threshold based on the chip-based dPCR assay targeting *sxtA4* to determine whether shellfish harvesting should be permitted in farms. As shown in Figure 6a, cell abundance estimated by the dPCR assay in field samples was divided into two groups on the basis of the traditional regulatory threshold of >80 μg PST 100 g^−1^ shellfish tissue (Group A) or a PST content below this value (Group B). Additionally, sanitary regulation for shellfish harvesting is often continued, despite a PST content of < 80 μg 100 g^−1^ shellfish tissue as measured by MBA, owing to an excess of PST in the area within a few days or a week. Considering these cases, we assigned samples to two additional groups based on the estimated cell numbers obtained by the dPCR assay, i.e., shellfish harvesting at the station is continuously regulated despite a lack of detectable PST (Group C) or shellfish harvesting is permitted (Group D). The mean abundance values in Groups A and C were 1961 (n = 24) and 1484 cells L^−1^ (n = 35), respectively, and in Groups B and D, in which shellfish harvesting was permitted, both showed a maximum concentration of 538 cells L^−1^ (n = 58 and n = 48, respectively). Given these results, we obtained an optimal regulatory threshold of 538 cells L^−1^ for the chip-based dPCR assay targeting *sxtA4*. To confirm the acceptability of the cell regulatory threshold, each of the four locations in which samples were collected during March and April from 2018 to 2021 were divided according to a threshold abundance of 538 cells L^−1^. We then compared the spatial distribution of *A*. *pacificum* with the regulated area for shellfish harvesting based on the PST threshold at the time reported by the KNIFS. 

In March–April 2018, the spatial distribution of samples with >538 cells L^−1^ corresponded to the regulated area based on the PST threshold (indicated by a red background in Figure 6b). Similar patterns were also observed in 2019, except at Jinhae (26 March), where cells were not detected by microscopy, although only two data sets were evaluated owing to a lack of sampling and no events in April 2019. In contrast, the distribution of *A*. *pacificum* with concentrations of > 538 cells L^−1^ in Jinhae-Masan Bay in March and April 2020 was not consistent with the regulated area based on PSP monitoring reports at the time, except for Makpo (18 March) and Jinhae and Gusan (6 April). Interestingly, cells in the genus *Alexandrium* were not detected by microscopy in the four locations during this period, except for Jinhae and Gusan (6 April), consistent with the results of the dPCR assay. In 31 March and 15 April, 2021, the distribution of samples with >538 cells L^−1^ at the stations corresponded to the red area, indicating the regulation of harvesting based on the PST threshold, except for Neungpo (31 March). In contrast, the spatial distribution of cells on 23 March and 29 April 2021 differed from the results of the dPCR assay and PST monitoring. For the station in which the PST content did not correspond to the cell abundance, microscopy results were consistent with the dPCR assay results. Taken together, the average similarity between the monitoring of *A*. *pacificum* based on the cell threshold for the chip-based dPCR targeting *sxtA4* and PST monitoring reports based on the PST threshold in March and April from 2018 to 2021 was 62.5%, with a maximum similarity of 91.7%.

**Figure 7:**
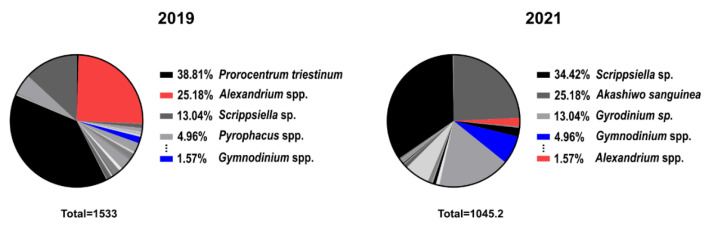
Community composition of Dinophyceae in Jinhae-Masan Bay estimated by direct counting of phytoplankton in field samples collected in March and April of 2019 and 2021.

**Figure 8:**
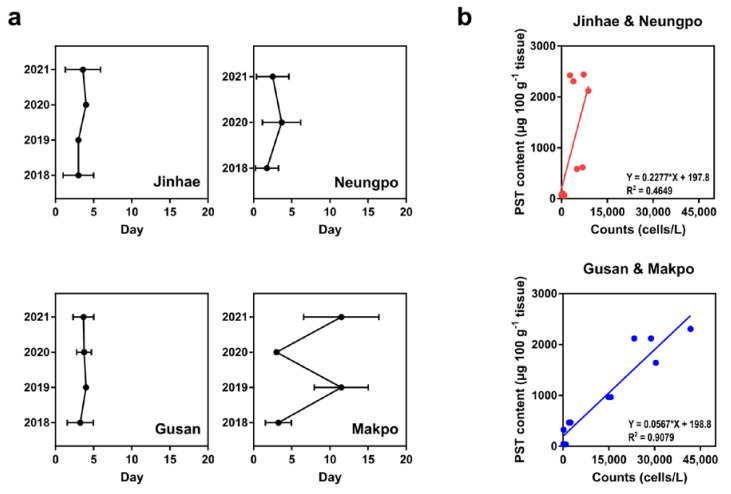
Factors affecting the establishment of a cell threshold value for *A*. *pacificum* to determine whether shellfish harvesting should be permitted at a particular station based on chip-based digital PCR (dPCR) targeting *sxtA4*. (**a**) Time interval between measurements of cell abundance estimated by the dPCR assay and PST content in shellfish tissue quantified by a mouse bioassay. Day 0 indicates the date that cells were detected by the dPCR assay, and the intervals for the detection of PST content are reported from Day 0 for Jinhae, Neungpo, Gusan, and Makpo; (**b**) relationships between cell abundance and PST content at each station according to geographical conditions, defined as offshore (Jinhae and Neungpo) and inshore (Gusan and Makpo). Factors were analyzed by comparing results of dPCR assays in this study with those of mouse bioassays reported by the KNIFS for March and April from 2018 to 2021.

Possible explanations for the 62.5% similarity are described here. The composition of dinoflagellates in Jinhae-Masan Bay, as determined by direct counting of phytoplankton in field samples, was significantly different in samples collected in March and April of 2019 than in those collected in 2021 (Figure 7). In 2019, the relative proportions of *Alexandrium* spp. and *Gymnodinium* spp. in the total population were 25.18% and 1.57%, respectively, compared with 1.57% and 4.96%, respectively, in 2021. This indicated that the occurrence of *Alexandrium* spp. in Jinhae-Masan Bay decreased from 2019 to 2021. The production of PST by *G. catenatum* in Korea has been reported since 1996 [41], and the toxicity of isolates of *G*. *catenatum* collected from the southern coastal waters in Korea has been investigated by Park et al. [42]. Although outbreaks of PST caused by *G*. *catenatum* in natural conditions have not been reported to date in Korea, additional studies of this species are required to prevent and control future blooms, in accordance with changes in the dinoflagellate community in Jinhae-Masan Bay from 2019 to 2021. Moreover, according to Shin et al. [12] and Baek et al. [43], *A. catenella*, one of the causative species of PSP outbreaks in Korean coastal waters, showed high germination success and toxicity below 15 °C, as well as a favorable growth of the cells at 10–15 °C, suggesting that early spring blooms in Jinhae-Masan Bay could be derived from *A*. *catenella*. To establish the dPCR assay for the prediction of PST-producing blooms in Korea, a detection method for *A*. *catenella* and *G*. *catenatum*, targeting PST biosynthesis genes, should be developed. Moreover, there could be a lag period between the detection of cells in field samples by the dPCR assay and the detection of PST in shellfish by the MBA because algal-derived toxins accumulate in shellfish tissues by filter-feeding activity [44]. We calculated the time interval from the date that *A*. *pacificum* cells were detected by the dPCR assay targeting *sxtA4* to the date that PSTs were detected in shellfish tissues at each station in March and April of 2018 to 2021. The time intervals at the four localities were 3.4 ± 1.8 (Jinhae), 3.6 ± 1.2 (Gusan), 6.5 ± 4.9 (Makpo), and 2.6 ± 1.9 (Neungpo) days (Figure 8a). In particular, the time interval of Makpo varied substantially, with estimates of 3.3 (2018), 11.5 (2019), 3.0 (2020), and 11.5 (2021) days. Time intervals longer than 3 days would affect the similarity rate between monitoring results for *A*. *pacificum* by the dPCR assay targeting *sxtA4* and PST monitoring based on PST contents in shellfish tissues in Jinhae-Masan Bay. Moreover, we analyzed relationships between cell abundance and the PST content at inshore and offshore locations for a linear regression analysis. The ratios of the detected PST content to the cell abundance in Jinhae and Neungpo (offshore) were higher than those for Gusan and Makpo (inshore) (Figure 8b). Although the linear regressions for both offshore and inshore values were based on small sample sizes (n = 13 each), the geographical conditions might affect the optimization of the cell regulatory threshold for the causative species of PST-producing blooms in Korea to determine whether shellfish harvesting should be permitted based on the dPCR assay. To establish a more precise cell threshold value for the molecular detection assay, further studies are required.

## 4. Conclusions

In Jinhae-Masan Bay, Korea, PST-producing blooms are mostly caused by *A*. *pacificum*. In this study, we established a method for quantifying *A*. *pacificum* cells using a chip-based dPCR targeting the *sxtA4* gene, which is involved in STX biosynthesis, and evaluated the practical application of the assay for long-term monitoring. The regulatory threshold for PSTs (80 μg 100 g^−1^ shellfish tissue) determined by MBAs was comparable with the cell concentrations of *A. pacificum* above 500 cells L^−1^, as measured using the dPCR assay. The consistency between results based on each detection threshold averaged 62.5% overall from 2018 to 2021, reaching a maximum of 91.7% from 2018 to 2019. To apply the dPCR assay for the prevention of potential PST-producing blooms in Jinhae-Masan Bay, a method for detecting other causative species, such as *A*. *catenella* and *G*. *catenatum*, based on PST biosynthesis genes should be developed. Although further studies are required to improve the accuracy of the dPCR assay, the newly established molecular detection method provides a complementary approach to traditional PST monitoring by MBA for the early detection and prediction of toxic dinoflagellate blooms in the southern coastal waters of Korea.

## 5. Materials and Methods

### 5.1. Strain Setup

The Masan strain of *A. pacificum* was isolated from Jinhae-Masan Bay in the southern coastal waters of Korea in May 2017. Samples were placed in six-well culture plates and a clonal culture of *A*. *pacificum* was established by five serial single-cell isolations aided by a dissecting microscope (SZX10; Olympus, Tokyo, Japan). The cells were grown in F/2 medium at 21 °C, with continuous illumination at 65 μmol photons m^−2^ s^−1^. After sufficient growth, the dense culture was transferred to 50, 250, and 500 mL polycarbonate (PC) bottles. The monoclonal culture of *A*. *pacificum* was subcultured monthly in fresh F/2 media, maintaining a cell density of at least 8.0 × 10^3^ cells mL^−1^.

### 5.2. DNA Extraction and PCR

Genomic DNA (gDNA) was extracted from cultures of *A*. *pacificum* with a density of 1.5 × 10^4^ cells mL^−1^ and field samples. Cells were harvested by centrifugation at 18,396 × *g* for 3 min, and the pellets were immediately stored at −80 °C until gDNA extraction. Preserved cell pellets and field samples collected on microporous membranes were homogenized in phosphate-buffered saline (PBS) before DNA extraction. The gDNA was extracted using the AccuPrep Genomic DNA Extraction Kit (BIONEER, Daejeon, Korea), following the manufacturer’s instructions. 

The clonal cultures were genotyped based on ribosomal DNA (rDNA) sequence analysis using universal eukaryotic primers [45,46,47]. PCR mixtures comprised 5 µL of 10× F-Star Taq Reaction Buffer with 1 µL of 10 mM dNTPs, 0.02 μM primer pairs (Table 1), 0.25 μL of 5 U μL^−1^ BioFACT™ F-Star Taq DNA Polymerase (BioFACT Co., Ltd., Daejeon, Korea), 38.75 µL of UltraPure™ DNAse/RNAse-Free Distilled Water (Invitrogen, Carlsbad, CA, USA), and 3 µL of the template DNA. PCR was performed using the Eppendorf Master Cycler PCR machine (Eppendorf, Hamburg, Germany) under the following thermal cycling conditions: pre-denaturation at 94 °C for 5 min; 40 cycles at 95 °C for 30 s, the selected annealing temperature (AT) for 30 s, and 72 °C for 1 min; with a final extension at 72 °C for 10 min. The AT of the primers was optimized using gradient PCR. PCR products were purified using the AccuPrep PCR Purification Kit (BIONEER) and confirmed by Sanger sequencing and alignment using BLAST from the National Center for Biotechnology Information (NCBI).

### 5.3. Species Identification

Morphological characterization of *Alexandrium* cells at the genus level was performed using an Olympus BX 53 microscope and images were obtained using a DP73 digital camera system (Olympus, Tokyo, Japan). For molecular characterization, cells of the Masan strain of *Alexandrium* were taxonomically identified by LSU rDNA phylogenetic analysis. LSU rDNA sequences of the isolated cells were amplified by conventional PCR using universal primers, and compared with the LSU sequences of 42 relatives in the *A. tamarense* species complex, three strains of *A*. *minutum*, and two strains of *Dinophysis acuminata* used as outgroups, obtained from GenBank. The sequence data were aligned using the SILVA alignment service [48]. A phylogenetic tree based on the aligned sequences was constructed using the ML algorithm and MEGA7 [49]. Bootstrap values were calculated using 1000 replicates with the Jukes–Cantor model. The ITS regions of rDNA in the cells were identified by conventional PCR with species-specific primers for *A*. *pacificum* and *A*. *catenella,* as reported by Lee et al. [14].

### 5.4. Quantification of the sxtA4 Gene by Chip-Based dPCR and qPCR

Putative *sxtA4* genomic sequences of the Masan strain of *A*. *pacificum* were amplified by PCR using previously reported primers [38], and verified by Sanger sequencing and BLASTx. For chip-based dPCR, the primer and probe set targeting *sxtA4* was designed using Primer3 (https://bioinfo.ut.ee/primer3-0.4.0/primer3/, accessed on 12 February 2019); the sequences are presented in Table 1.

A Clarity™ Digital PCR System was used to quantify the number of gene copies within a sample following the manufacturer’s protocols (JN Medsys, Singapore). Reaction mixtures contained 7.5 µL of qPCRBIO Probe Mix no-ROX (2×) (PCR Biosystems, London, England), 0.75 µL of JN Solution (20×), 0.4 µL of 10 µM primer pairs, 0.5 µL of 10 µM TaqMan probe, 2.45 µL of UltraPure™ DNAse/RNAse-Free Distilled Water (Invitrogen), and 3 µL of the template DNA in a total volume of 20 µL. In the assay, UltraPure™ distilled water was used as a negative control. Each PCR mixture was subdivided into 10,000 partitions in the chip using the Clarity™ Auto Loader and Sealing Enhancer following the manufacturer’s instructions, and then subjected to thermal cycling under the following conditions: initial denaturation at 95 °C for 5 min; 40 cycles of 95 °C for 50 s and the selected AT for 90 s; with a final extension at 72 °C for 5 min. As an endpoint analysis, fluorescent signals from each partition were detected concurrently using the Clarity™ Reader after PCR amplification. The AT of the designed primers was optimized by gradient dPCR. Under the optimized conditions, we tested the specificity of the specific primers for the dPCR assay using *A*. *pacificum*, *A*. *catenella*, *A*. *minutum*, and *G*. *catenatum*, which were all laboratory cultured species.

The number of gene copies μL^−1^ of sample was determined using Clarity software based on Poisson statistics and Equation (1), where D is the dilution factor, Vp is the partition volume, p is the number of positive partitions, and N is the number of total partitions [50,51,52]. The *T* value was used to calculate the number of gene copies per cell using Equation (2), where Vr is the reaction volume, Vs is the original sample volume, Vt is the volume of template DNA, and c is the known number of cells [36].

(1) $$T=-\frac{D}{Vp}\times \ln(-\frac{p}{N})$$

(2) $$gene\;copies/cell=T\times Vr\times \frac{Vs}{Vt}\times \frac{1}{c}$$

A qPCR assay was performed to estimate the abundance of *A*. *pacificum* with specific primers for *sxat4* using a PCRmax Eco 48 Real-time PCR System (Staffordshire, UK). PCR mixtures contained 10 µL of qPCRBIO Probe Mix no-ROX (2×) (PCR Biosystems, London, England), 1 µL of 10 µM primer pairs, 0.5 µL of 10 µM TaqMan probe, 4.5 µL of UltraPure™ DNAse/RNAse-Free Distilled Water (Invitrogen), and 3 µL of the template DNA in a final volume of 20 µL. Thermal cycling was performed as follows: initial denaturation at 95 °C for 3 min; 40 cycles of 95 °C for 10 s and extension at 60 °C for 30 s. The amplification efficiency was evaluated from the standard curve calculated by the threshold cycle (Ct) values.

### 5.5. Field Sampling

**Figure 9:**
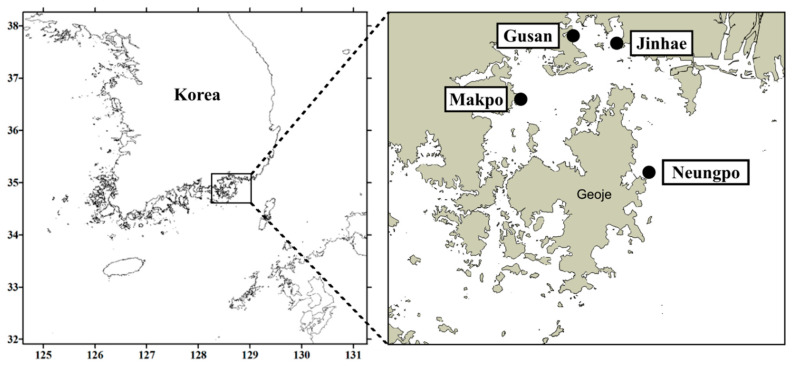
Location of sampling stations, including Jinhae, Gusan, Makpo, and Neungpo, in Jinhae-Masan Bay in the southern coastal waters of Korea.

Field seawater samples were collected from four locations in Jinhae-Masan Bay, Korea: Jinhae (JH, 35°05′58.9″ N 128°42′05.5″ E), Gusan (GS, 35°07′11.4″ N 128°35′50.8″ E), Makpo (MP, 35°01′17.7″ N 128°29′59.4″ E), and Neungpo (NP, 34°53′44.4″ N 128°45′06.5″ E) in March and April from 2018 to 2021 (Figure 9). To obtain environmental DNA from the field samples, 100–200 mL of seawater at each location was filtered through a GF/C glass microfiber filter and stored at −80 °C until DNA extraction. The gDNA was extracted from the frozen samples and subjected to *sxtA4* gene copy number analysis to determine the abundance of *A*. *pacificum* using chip-based dPCR and qPCR assays. Samples containing living cells were fixed in Lugol’s iodine solution, and *Alexandrium* cells identified at the genus level were counted directly using a Sedgwick Rafter counting (SRC) chamber aided by a dissecting microscope (SZX10; Olympus).

### 5.6. Comparative Analysis

The cell abundance estimated by chip-based dPCR targeting *sxtA4* in field samples collected from Jinhae-Masan Bay in March and April from 2018 to 2021 was compared to the total PST content in shellfish tissues collected from the same stations and time points (presented in PSP monitoring reports by the KNIFS). The PST content was analyzed using an MBA and is conducted annually for the prevention of PSP outbreaks by the KNIFS [13]. According to the Korean Ministry of Food and Drug Safety (KMFDS) in 2015, the PST analysis was carried out by intraperitoneally injecting 1 mL of the prepared mixture extracted with HCl into ICR (CD-1) female mice weighing 19–21 g (20 ± 1 g) [13]. After monitoring neurotoxic shellfish poisoning symptoms and mouse survival times, the PST content in the sample was calculated on the basis of Sommer’s table.

Correlations between cell abundance in environmental samples estimated by chip-based dPCR and other detection methods (qPCR and microscopy) were analyzed using GraphPad Prism version 7.0 (GraphPad, San Diego, CA, USA). The cell concentrations converted from *sxtA4* gene copy numbers in each detection method were plotted on a logarithmic scale. Samples collected at the four locations (JH, GS, MP, and NP) in March and April from 2018 to 2021 were separated into groups based on a threshold cell abundance of 538 cells L^−1^, estimated by the chip-based dPCR assay targeting *sxtA4*. In Jinhae-Masan Bay, the spatial distribution of *A*. *pacificum* at concentrations above and below 538 cells L^−1^ was compared with regulated areas for shellfish harvesting based on the PST threshold reported by the KNIFS. The PST content was plotted against cell counts, as determined by the chip-based dPCR assay, at the four locations and the linear regression calculated using GraphPad Prism.

## Figures and Tables

**Table 1:** Primers used in PCR, sequencing, qPCR, and dPCR.

Primer Name	Primer Region	Sequence (5′–3′)	Reference
EukA	Forward, SSU	AACCTGGTTGATCCTGCCAG	[45]
G18R	Reverse, SSU	GCATCACAGACCTGTTATTG	[46]
570F	Forward, SSU	GTAATTCCAGCTCCAATAGC	[47]
EukB	Reverse, SSU	TGATCCTTCTGCAGGTTCACCTAC	[45]
ITSF2	Forward, ITS	TACGTCCCTGCCCTTTGTAC	[46]
LSUB	Reverse, LSU	ACGAACGATTTGCACGTCAG	[46]
LSU500R	Reverse, LSU	CCCTCATGCTACTTGTTTGC	[46]
LSU500F	Forward, LSU	GCAAACAAGTACCATGAGGG	[46]
ITSFR2	Reverse, ITS	TCCCTGTTCATTCGCCATTAC	[46]
Sxt007	Forward, *sxtA4*	ATGCTCAACATGGGAGTCATCC	[38]
Sxt008	Reverse, *sxtA4*	GGGTCCAGTAGATGTTGACGATG	[38]
SxtA4MAPF	Forward, *sxtA4* of the Masan strain of *A*. *pacificum*	GCTGGACTACGCGGAGAA	this study
SxtA4MAPR	Reverse, *sxtA4* of the Masan strain of *A*. *pacificum*	GGCGAATTGAACGCCTTGCT	this study
SxtA4MAPP	Probe, *sxtA4* of the Masan strain of *A*. *pacificum*	AACATCATCTACGCCGGGCAGC	this study

## Data Availability

Not applicable.
